# Computational Studies on the Potency and Selectivity of PUGNAc Derivatives Against GH3, GH20, and GH84 β-N-acetyl-D-hexosaminidases

**DOI:** 10.3389/fchem.2019.00235

**Published:** 2019-04-12

**Authors:** Lili Dong, Shengqiang Shen, Yefei Xu, Leng Wang, Ruirui Feng, Jianjun Zhang, Huizhe Lu

**Affiliations:** Department of Applied Chemistry, College of Science, China Agricultural University, Beijing, China

**Keywords:** PUGNAc derivatives, β-N-acetyl-D-hexosaminidases, selectivities, molecular docking, molecular dynamics simulations, binding free energy, free energies decomposition

## Abstract

β-N-acetyl-D-hexosaminidases have attracted significant attention due to their crucial role in diverse physiological functions including antibacterial synergists, pathogen defense, virus infection, lysosomal storage, and protein glycosylation. In particular, the GH3 β-N-acetyl-D-hexosaminidase of *V. cholerae* (VcNagZ), human GH20 β-N-acetyl-D-hexosaminidase B (HsHexB), and human GH84 β-N-acetyl-D-hexosaminidase (hOGA) are three important representative glycosidases. These have been found to be implicated in β-lactam resistance (VcNagZ), lysosomal storage disorders (HsHexB) and Alzheimer's disease (hOGA). Considering the profound effects of these three enzymes, many small molecule inhibitors with good potency and selectivity have been reported to regulate the corresponding physiological functions. In this paper, the best-known inhibitors PUGNAc and two of its derivatives (N-valeryl-PUGNAc and EtBuPUG) were selected as model compounds and docked into the active pockets of VcNagZ, HsHexB, and hOGA, respectively. Subsequently, molecular dynamics simulations of the nine systems were performed to systematically compare their binding modes from active pocket architecture and individual interactions. Furthermore, the binding free energy and free energy decomposition are calculated using the MM/GBSA methods to predict the binding affinities of enzyme-inhibitor systems and to quantitatively analyze the contribution of each residue. The results show that PUGNAc is deeply-buried in the active pockets of all three enzymes, which indicates its potency (but not selectivity) against VcNagZ, HsHexB, and hOGA. However, EtBuPUG, bearing branched 2-isobutamido, adopted strained conformations and was only located in the active pocket of VcNagZ. It has completely moved out of the pocket of HsHexB and lacks interactions with HsHexB. This indicates why the selectivity of EtBuPUG to VcNagZ/HsHexB is the largest, reaching 968-fold. In addition, the contributions of the catalytic residue Asp253 (VcNagZ), Asp254 (VcNagZ), Asp175 (hOGA), and Asp354 (HsHexB) are important to distinguish the activity and selectivity of these inhibitors. The results of this study provide a helpful structural guideline to promote the development of novel and selective inhibitors against specific β-N-acetyl-D-hexosaminidases.

## Introduction

β-N-acetyl-D-hexosaminidases (EC 3.2.1.52) are widely distributed glycosidases, which can catalyze the cleavage of terminal non-reducing β-O-linked N-acetyl-D-glucosamine (GlcNAc) or N-acetyl-D-galactosamine (GalNAc) residues. Based on similarities of amino acid structure and sequence in the CAZy database (http://www.cazy.org), these enzymes are classified into three glycoside hydrolase (GH) families: GH3, GH20, and GH84 (Henrissat and Davies, 1997; Cantarel et al., 2009). These families are involved in diverse physiological functions including antibacterial synergists (Stubbs et al., 2007), pathogen defense (Liu et al., 2014), virus infection (Liu et al., 2014), lysosomal storage (Liu et al., 2018), and protein glycosylation (Hart et al., 2007; Butkinaree et al., 2010). GH3 β-N-acetyl-D-hexosaminidase, also named NagZ, removes GlcNAc from GlcNAc-1,6-anhydromuramoyl-peptides and produces 1,6-anhydroMurNAc peptides in many Gram-negative bacteria (Cheng et al., 2000; Stubbs et al., 2007; Balcewich et al., 2009). Consequently, NagZ can induce AmpC β-lactamase expression and regulate peptidoglycan (PG) recycling. Once the activity of NagZ is blocked, suppression of AmpC-mediated β-lactam resistance will occur (Stubbs et al., 2007, 2013; Asgarali et al., 2009). Thus, this is a promising strategy to target the cytosolic GH NagZ with small molecule inhibitors. GH20 β-N-acetyl-D-hexosaminidases are widely distributed and versatile enzymes in nature. In humans, deficiency of this enzyme can cause the storage of GM2 ganglioside in neuronal lysosomes, which will result in severe neurodegenerative disorders such as Tay-Sachs and Sandhoff diseases (Mahuran, 1999; Platt, 2014; Liu et al., 2018). In insects and fungi, GH20 β-N-acetyl-D-hexosaminidase is generally responsible for the degradation of chitin during growth (Merzendorfer and Zimoch, 2003; Chen et al., 2017). GH84 β-N-acetyl-D-hexosaminidase in metazoan cells removes *O*-linked *N*-acetylglucosamine from both nucleus and cytoplasmic proteins, which is a central physiological process of the regulatory function of O-GlcNAcylation. Dysregulation of human O-GlcNAcylation has been implicated in several diseases, such as cancer, type II diabetes, and Alzheimer's disease (Issad et al., 2010; Singh et al., 2015; Frenkel-Pinter et al., 2017).

**Figure 1:**
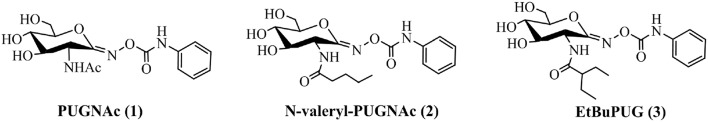
Non-selective and selective inhibitors of GH3, GH20 and GH84 β-N-acetyl-D-hexosaminidases.

Inhibiting specific GH3, GH20, and GH84 β-N-acetyl-D-hexosaminidases has been considered as a promising approach to regulate corresponding physiological functions, thus avoiding the side effects caused by the undesired inhibition of similar enzymes. A number of small molecule inhibitors have been reported, which include PUGNAc(**1**) (Horsch et al., 1991), NAG-Thiazoline (Knapp et al., 1996), GlcNAcstatins (Dorfmueller et al., 2009, 2010), iminocyclitols (Ho et al., 2010), TMG-chitotriomycin (Usuki et al., 2008), pyrimethamine (Maegawa et al., 2007), and naphthalimides (Tropak et al., 2007; Guo et al., 2013; Shen et al., 2018). Among these inhibitors, PUGNAc is the best-known molecule. This compound was shown to potentially inhibit GH3 β-N-acetyl-D-hexosaminidase from *V. cholerae* (VcNagZ), human GH20 β-N-acetyl-D-hexosaminidase (HsHexB), and human GH84 O-GlcNAcase (hOGA), achieving K_i_ values of 48 nM (Stubbs et al., 2007), 36 nM (Macauley et al., 2005), and 46 nM (Macauley et al., 2005), respectively. The structural basis for the potency of PUGNAc lies in the sp^2^-hybridized carbon at the C-1 position, which mimics the conformation of the relatively planar oxocarbenium ion-like transition state (Whitworth et al., 2007; Macauley et al., 2008; He et al., 2011). Furthermore, the oxime substituent further contributes to the potency of PUGNAc by forming additional hydrogen binding energy (Whitworth et al., 2007; Macauley et al., 2008; He et al., 2011). Even though PUGNAc offers these benefits of potency, it lacks selectivity. To improve the selective inhibition against GH3 NagZ, here, a series of 2-acyl modified derivatives of PUGNAc were synthesized. For instance, N-valeryl-PUGNAc(**2**) showed increased selectivity for GH3 VcNagZ (K_i_ = 0.33 μM) (Stubbs et al., 2007) over human GH20 HsHexB (K_i_ = 220 μM) (Stubbs et al., 2006) and GH84 hOGA (K_i_ = 40 μM) (Stubbs et al., 2006). EtBuPUG(**3**) was found to be the most selective inhibitor of GH3 VcNagZ over human GH20 HsHexB and GH84 hOGA, achieving selectivity ratios of 109 and 1,000 (Figure 1 and Table 1) (Stubbs et al., 2007).

**Table 1:** Inhibition constants K_i_ (μM) and selectivity of PUGNAc derivatives against VcNagZ, HsHexB, and hOGA.

**Compound**	**K**_****i****_ **(μM)**			**Selectivity ratio**		
	**VcNagZ**	**HsHexB**	**hOGA**	**K_**i (HsHexB)**_/K_**i (VcNagZ)**_**	**K_**i (hOGA)**_/K_**i (VcNagZ)**_**	**K_**i (HsHexB)**_/K_**i (hOGA)**_**
**1**	0.048^a^	0.036^b^	0.046^b^	0.75	1.0	0.78
**2**	0.33^a^	220^c^	40^c^	667	121	5.5
**3**	3.1^a^	>3000^a^	337^a^	>968	109	>8.9

a *Results determined previously (Stubbs et al., 2007)*.

b *Results determined previously (Macauley et al., 2005)*.

c *Results determined previously (Stubbs et al., 2006)*.

In an effort to study how GH3, GH20, and GH84 β-N-acetyl-D-hexosaminidases could recognize PUGNAc and its derivatives at atomic level, several crystal structures of enzyme-inhibitor complexes were reported and analyzed (Mark et al., 2003; Dorfmueller et al., 2006, 2009; Rao et al., 2006; He et al., 2011; Liu et al., 2011; Sumida et al., 2012; Slamova and Kren, 2013; Robb et al., 2015; Elsen et al., 2017; Roth et al., 2017; Vadlamani et al., 2017). The crystal structures of GH3 VcNagZ bound to PUGNAc (PDB code: 2OXN) (Stubbs et al., 2007), N-valeryl-PUGNAc (PDB code: 3GSM) (Balcewich et al., 2009), and GH3 β-N-acetyl-D-hexosaminidase from *B. cenocepacia* (BcNagZ) bound to both PUGNAc (PDB code: 5UTQ) and EtBuPUG (PDB code: 5UTP) have been reported (Vadlamani et al., 2017). The results showed that the remarkable flexibility of NagZ enzymes enabled them to accommodate different conformations in response to various inhibitors, and displacement of the catalytic loop by PUGNAc derivatives considerably opened the entrance to the active pockets. The crystal structures of GH20 β-N-acetyl-D-hexosaminidases from *Paenibacillus sp*. (PsHex) (Sumida et al., 2012) and *Ostrinia furnacalis* (OfHex1) (Liu et al., 2011) bound to PUGNAc (PDB code: 3SUT and 3OZP, respectively) showed that the sensitivities of GH20 β-N-acetyl-D-hexosaminidases to PUGNAc were determined by the size of the active pocket. Furthermore, the crystal structures of GH84 β-N-acetyl-D-hexosaminidase from human (hOGA), which was bound to the PUGNAc-type inhibitor (PDB code:5M7T), showed that a particular dimer with intertwined helical-bundle domains that leads to the formation of the substrate-binding site, and high potency inhibitors could bind both the active site and the unique surrounding peptide-binding site (Roth et al., 2017).

These studies on the crystal structures of GH3, GH20, and GH84 β-N-acetyl-D-hexosaminidases in complexes with PUGNAc and its derivatives partially clarified the binding mechanisms of related inhibitors with different GH β-N-acetyl-D-hexosaminidases. Nevertheless, the crystal structure of the complexes of GH20 and GH84 β-N-acetyl-D-hexosaminidases bound to N-valeryl-PUGNAc and EtBuPUG has not been reported to date. Furthermore, few in-depth studies have been published about the dynamic changes of GH3, GH20, and GH84 β-N-acetyl-D-hexosaminidases bound to PUGNAc, N-valeryl-PUGNAc, and EtBuPUG. Such dynamic mechanisms could be closer to the models of inhibitor binding with target enzymes *in vivo*, thus it may provide interesting to investigate the details of the protein–ligand interaction (Anderson et al., 2005; Zhang et al., 2005; Falck et al., 2008; Vergara-Jaque et al., 2012;Soumana et al., 2016).

Given the profound physiological functions of GH3 VcNagZ, GH20 HsHexB, and GH84 hOGA on the regulation of biological processes, in this paper, PUGNAc, N-valeryl-PUGNAc, and EtBuPUG (which possess different inhibitory potency against these three enzymes) were selected as model compounds. Then, possible inhibitory modes of selected inhibitors against the specific β-N-acetyl-D-hexosaminidases were investigated using molecular docking, molecular dynamics (MD) simulations. Finally, comparing these established binding patterns, clarifies the reasons for both the potency and selectivity of PUGNAc and its derivatives against GH3, GH20, and GH84 β-N-acetyl-D-hexosaminidases, which could further guide the discovery of highly effective and specific inhibitors.

## Materials and Methods

### Molecule Preparation and Optimization

PUGNAc, N-valeryl-PUGNAc, and EtBuPUG molecules were constructed using the Sketch Molecule module of SYBYL 7.3 software package (Horsch et al., 1991; Stubbs et al., 2006, 2007; Tripos Associates, 2006). To achieve corresponding low energy conformations, these compounds were then fully optimized at the B3LYP/6-31G(d) basis set, using density functional theory (DFT) in Gaussian 16 (Frisch et al., 2016). The optimized conformation was used for the subsequent docking study.

### Molecular Docking

Molecular docking can effectively predict and characterize predominant binding sites of a ligand located in the protein. In this study, the X-ray crystal complex structures of GH3 VcNagZ (PDB code: 5UTQ) (Vadlamani et al., 2017), GH20 HsHexB (PDB code: 1NP0) (Mark et al., 2003), and GH84 hOGA (PDB code: 5M7T) (Roth et al., 2017) were obtained from the Protein Data Bank (http://www.rcsb.org/pdb/home/home.do). These were selected as the starting structure for docking, using the Surflex-Dock program of the SYBYL 7.3 software package (Tripos Associates, 2006). Surflex-Dock uses an empirically derived scoring function that is based on the binding affinities of protein-ligand complexes and on their respective X-ray structures. Several preliminary steps were required before docking, such as removing water molecules and adding hydrogens. In the following, the ligand mode was applied to generate the protomol, a suitable putative ligand pose, which was then used for the docking of the small molecules PUGNAc, N-valeryl-PUGNAc, and EtBuPUG. During the docking studies, ring flexibility was considered, and default settings were chosen for the remaining parameters. The binding affinities of the protein–ligand complexes were obtained with the Surflex scoring, which considers hydrophobic, polar, repulsive, entropic entropic, and solvated effects (Welch et al., 1996).

### MD Simulations

The enzymes of VcNagZ, HsHexB, and hOGA and three compounds of PUGNAc, N-valeryl-PUGNAc, and EtBuPUG, constituted nine systems, which were used for the MD calculations in the Amber14 program (Case et al., 2014). For each of the ligand–protein system simulations, the force field parameters for proteins and inhibitors were represented by AMBER03 (Duan et al., 2003) and GAFF (Wang et al., 2004) force field, respectively. Then, each complex structure was solvated in a truncated octahedral box in which TIP3P water molecules extended 10 Å from the complex. Counterions (Cl^−^ or Na^+^) were added to achieve electrostatic neutrality. Two successive energy minimizations were realized using the Sander module prior to MD simulations. First, the hydrogen atoms and water molecules were minimized with the first 2,500 steps of steepest descent and the last 2,500 steps of the conjugate gradient algorithm. Then, 2,500 cycles of the steepest-descent and 2,500 cycles of the conjugated gradient algorithm were used to minimize all atoms of the systems without restriction. Following minimization, these systems were gradually heated from 0 to 300 K in the NVT ensemble during 100 ps with a restrain force constant of 5 kcal/mol/Å^2^, which was applied to the backbone atom. Next, the 100 ps MD simulations with a 5 kcal/mol/Å^2^ restraint on the backbone atoms were carried out at a pressure of 1 atm and 300 K to equilibrate the density using Berendsen's barostat. These systems were then equilibrated at 300 K and pressure of 1 atm without any constraints. Finally, MD simulations of 20 ns were performed at a constant temperature of 300 K and a pressure of 1 atm employing the PMEMD module in Amber14 (Case et al., 2014). The SHAKE method (Ryckaert et al., 1977) was used to constrain hydrogen atoms and the time step was set to 2.0 fs. The particle mesh Ewald (PME) algorithm (Darden et al., 1993) was applied to treat the long-range electrostatic interactions with default cutoff of 8.0 Å under periodic boundary conditions.

### MM/GBSA Calculations

The coordinate trajectories were saved every 10 ps and stable conformations, that were generated from the last 2 ns of simulations, were applied to binding free energy calculations and analyses using MM/GBSA methods implemented in Amber14 (Case et al., 2014). In MM/GBSA, the binding free energy (ΔGbind) was calculated as follows:

(1) $$\begin{array}{l} \Delta {\text{G}}_{\text{bind}}={\text{G}}_{\text{complex}}-\left({\text{G}}_{\text{receptor}}+{\text{G}}_{\text{ligand}}\right) \end{array}$$

(2) $$\begin{array}{l} \Delta {\text{G}}_{\text{bind}}=\Delta {\text{E}}_{\text{MM}}+\Delta {\text{G}}_{\text{solvation}}-\text{T}\Delta \text{S} \end{array}$$

(3) $$\begin{array}{l} \Delta {\text{E}}_{\text{MM}}=\Delta {\text{E}}_{\text{internal}}+\Delta {\text{E}}_{\text{electrostatic}}+\Delta {\text{E}}_{\text{VDW}} \end{array}$$

(4) $$\begin{array}{l} \Delta {\text{G}}_{\text{solvation}}=\Delta {\text{G}}_{\text{sol}}^{\text{nonpolar}}+\Delta {\text{G}}_{\text{polarsol}} \end{array}$$

where *G*_complex_, G_receptor_, and G_ligand_ represent the free energy of complex, protein, and ligand, respectively. Δ*G*_bind_ was evaluated via the entropy term (TΔ*S*), the solvation energy term (Δ*G*_solvation_), and the gasphase interaction energy (Δ*E*_MM_) between inhibitors and receptors. ΔE_MM_ represents the gasphase interaction energy, which includes internal (Δ*E*_internal_), electrostatic (Δ*E*_electrostatic_), and van der Waals energies (Δ*E*_VDW_). Δ*G*_solvation_ accounts for the change of solvation energy that combines polar and nonpolar components of the desolvation free energy. TΔ*S* is the change of conformational entropy in response to ligand binding. The non-polar contribution was estimated via the solvent accessible surface area (SASA) using the LCPO method implemented in Amber (Weiser et al., 1999). The polar component of the desolvation energy was determined by the GB (igb = 2) of Onufriev et al. (2004). The solute and outer dielectric constants were set to 80 and 1, respectively. All energy components were performed for 2000 snapshots and extracted from the last 2 ns MD trajectories. Then, the energy decomposition was used to calculate the contribution of each residue of the systems to the total binding free energies using the MM/GBSA method in Amber14 (Case et al., 2014).

## Results and Discussion

### Molecular Docking of PUGNAc, N-Valeryl-PUGNAc, and EtBuPUG Into VcNagZ, HsHexB, and hOGA

**Figure 2:**
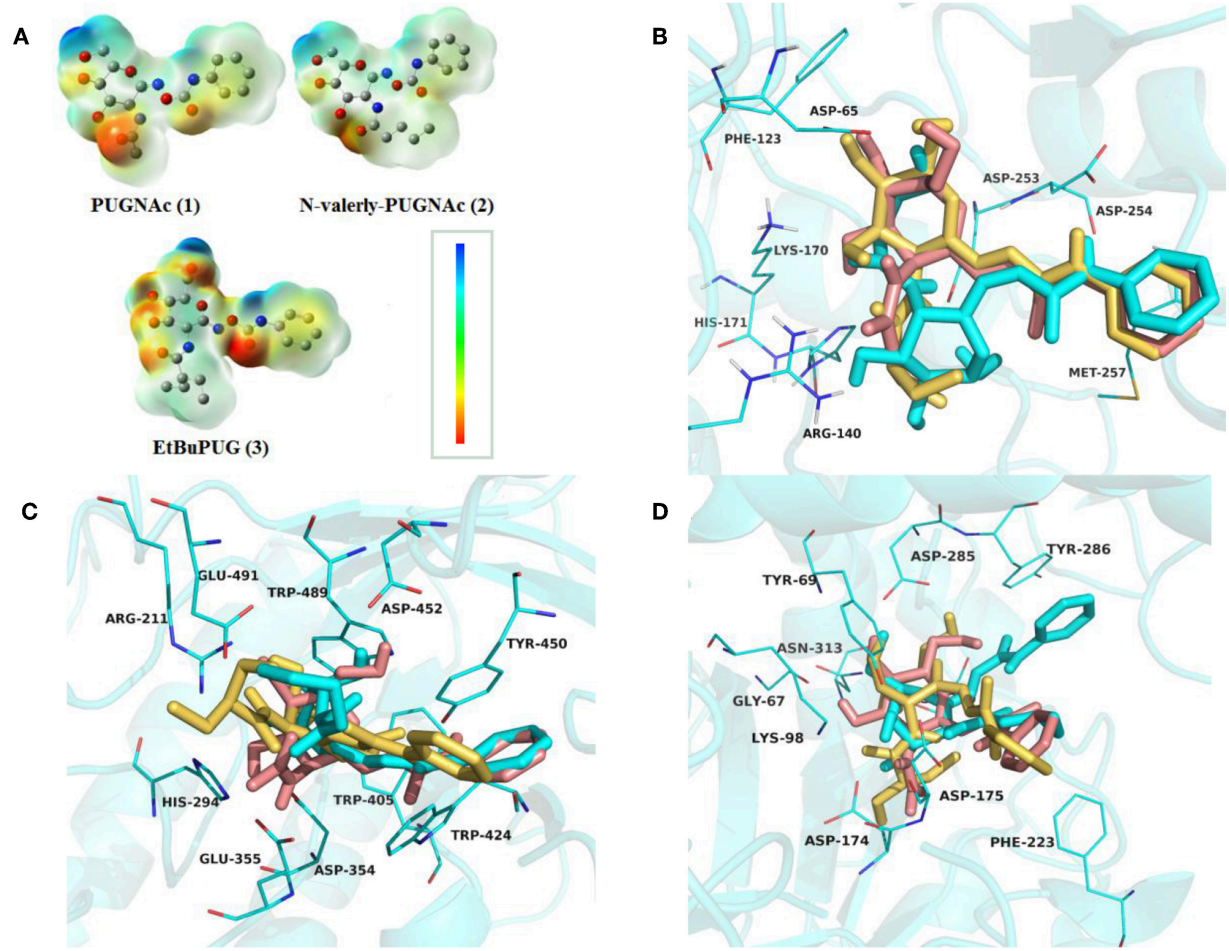
Representation of the optimized ligands PUGNAc(**1**), N-valeryl-PUGNAc(**2**) and EtBuPUG(**3**) using DFT calculations **(A)** and the sound docked conformation of PUGNAc derivatives in the active site of the VcNagZ **(B)**, HsHexB **(C)** and hOGA **(D)**, respectively. The charge from negative to positive was showed as different colors: from red to blue. The ligands PUGNAc, N-valeryl-PUGNAc and EtBuPUG are shown as sticks with pink, cyan and yellow orange color, respectively.

To investigate the basis for both the potency and selectivity of PUGNAc derivatives toward VcNagZ, HsHexB, and hOGA, the binding modes of PUGNAc, N-valeryl-PUGNAc, and EtBuPUG into these three enzymes were observed via molecular docking. Firstly, density functional theory (DFT) calculations at the B3LYP/6-31G (d) basis set were performed to optimize the PUGNAc derivatives using the Gaussian 16 program. The optimized molecules are shown in Figure 2A. Compared to PUGNAc and N-valeryl-PUGNAc, the oxygen on the C-6 in the pyranose ring of EtBuPUG was found to have become perpendicular to the paper plane. This phenomenon may be because the 2-isobutamido group of EtBuPUG is relatively larger and changes the conformation of its pyranose ring. Furthermore, a blue positive area was observed near the pyranose ring of these compounds, as well as a slightly yellow negative area close to benzene, which complemented the charge in the active pocket of the enzymes (Figure S1). Thus, good electrostatic interaction between small molecules and enzymes may be beneficial for protein-ligand binding. Subsequently, these three optimized inhibitors were docked into the active sites of VcNagZ, HsHexB, and hOGA, respectively. Among docking score results, total_score is expressed as the binding constant Log(Kd), where a high value of the total score represents a good binding ability. Crash indicates the degree of inappropriate penetration by the ligand into the protein and of interpenetration (self-clash) between ligand atoms (Pham and Jain, 2006). Crash scores close to 0 are favorable and beneficial for protein-ligand binding. Polar indicates the contribution of polar interactions to the total score. Similarity indicates the Surflex-Sim similarity between the scoring pose and the ligand (Jain, 2000).

As shown in Table 2, the total scores of compounds were consistent with the ranking of their experimental activities, and the similarity scores all exceeded 0.6, which indicated that the results of docking were trustworthy. Furthermore, the polar scores of these systems of PUGNAc combined with VcNagZ, HsHexB, and hOGA appeared to be very high, which indicates that PUGNAc have strong polar interactions with the three β-N-acetyl-D-hexosaminidases. The crash absolute value of EtBuPUG was largest, which may be because its bulky 2-acetamido substituent group leads to a relatively larger steric hindrance.

**Table 2:** Docking scores results predicted for the PUGNAc derivatives against β-N-acetyl-D-hexosaminidase.

**Compound**	**Total_Score**			**Crash**			**Polar**			**Similarity**		
	**VcNagZ**	**HsHexB**	**hOGA**	**VcNagZ**	**HsHexB**	**hOGA**	**VcNagZ**	**HsHexB**	**hOGA**	**VcNagZ**	**HsHexB**	**hOGA**
**1**	7.71	9.21	8.43	−1.20	−2.04	−1.47	4.97	6.36	5.12	0.69	0.67	0.72
**2**	6.75	5.15	5.33	−1.33	−1.12	−2.31	4.91	3.43	5.05	0.62	0.63	0.74
**3**	5.61	4.02	4.24	−2.40	−4.96	−4.12	4.53	4.32	4.98	0.66	0.65	0.80

The detailed binding modes of PUGNAc, N-valeryl-PUGNAc, and EtBuPUG toward VcNagZ, HsHexB and hOGA are demonstrated in Figures 2B–D. The pyranose ring of these three inhibitors was found to be bound to the−1 subsite (active pocket) of all the three enzymes and the benzene ring extends out from the active pocket. Figure 2B shows that the inhibitors are located well at the VcNagZ active site pocket and formed electrostatic, hydrophobic and hydrogen bond interactions with the residues Asp65, Arg140, Lys170, His171, Asp253, Asp254, and Met257, as previously reported (Vadlamani et al., 2017). Figure 2C shows the inhibitors bound to the active site of HsHexB and their interactions with residues Arg211, Glu355, Tyr450, Asp452, Tyr456, and Glu491. Figure 2D shows the binding modes between the inhibitors and hOGA, and small molecules interact with residues Gly67, Tyr69, Lys98, Asp174, Asp175, Phe223, Asn280, Asp285, Tyr286, Asn313, and Trp645. The above results indicated that the obtained docking sites are reliable and conducive to the development of molecular dynamics.

### MD Simulations

**Figure 3:**
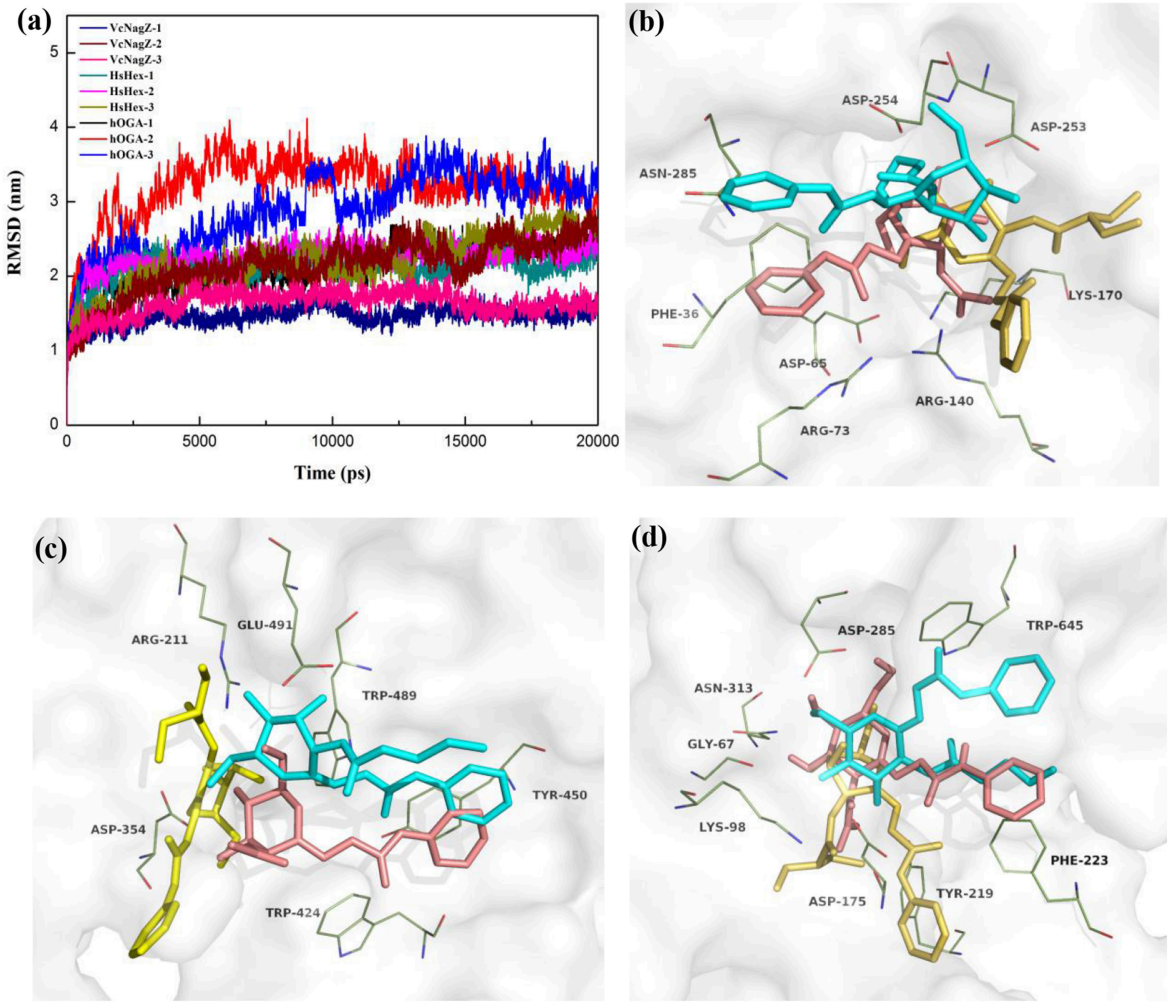
Predicted binding mechanism of PUGNAc(**1**), N-valeryl-PUGNAc(**2**) and EtBuPUG(**3**) with VcNagZ, HsHexB and hOGA, respectively, revealed by 20 ns MD simulations. **(a)** RMSD changes of nine simulated systems. **(b–d)** Binding modes of compounds **1**, **2**, and **3** with VcNagZ **(b)**, HsHexB **(c)**, and hOGA **(d)**, respectively, at 20 ns of MD simulations. The ligands **1**, **2**, and **3** are shown as sticks with pink, cyan and yellow-orange color, respectively. The residues that interact with the compounds are shown as lines with green.

In an effort to further investigate the appropriate interaction mode of PUGNAc, N-valeryl-PUGNAc, and EtBuPUG binding to VcNagZ, HsHexB, and hOGA, 20 ns MD simulations were conducted to further consider the flexibility of the whole protein and observe the dynamic binding mechanism of inhibitor-enzyme systems. As illustrated in Figure 3a, the dynamic convergences were all achieved at approximately 16 ns and the RMSD values of the backbone atoms were ultimately maintained at around 1.6–3.1 Å for the nine simulated systems. These data indicated that the complexes underwent appropriate conformational change (Figure 3a and Figures S2,S3).

Figures 3b–d and Figure S4 represent the superimposition of the conformations of the three inhibitors bound into VcNagZ, HsHexB, and hOGA, respectively, at 20 ns of MD simulations. Compared to N-valeryl-PUGNAc and EtBuPUG, PUGNAc is deeply-buried in the active pockets of these three enzymes. The conformation of N-valeryl-PUGNAc is similar to that of PUGNAc but it is buried shallower than PUGNAc. This may result in the activity of N-valeryl-PUGNAc being lower than that of PUGNAc (reduced activity by 7-, 6,111-, and 870-fold against VcNagZ, HsHexB, and hOGA than against PUGNAc). EtBuPUG bearing branched 2-isobutamido further made its volume larger and unstable to binding into the active pocket, which resulted in a reduced activity by about 10-fold against VcNagZ, HsHexB, and hOGA compared to N-valeryl-PUGNAc.

Figure S5 shows the two-dimensional (2D) representation of the binding modes of these compounds in the active pockets of VcNagZ, HsHexB, and hOGA, respectively. The results indicate that PUGNAc forms strong hydrogen bond interactions with the catalytic residues Asp253 (VcNagZ), Asp254 (VcNagZ), Asp354 (HsHexB), and Asp175 (hOGA). Moreover, the N-phenylcarbamate of PUGNAc also forms π-π stacking and hydrophobic interaction with the surrounding residues Phe36 (VcNagZ), Tyr450 (HsHexB), and Phe223 (hOGA). However, N-valeryl-PUGNAc and EtBuPUG hardly interacted with the corresponding catalytic residues of these enzymes. Furthermore, the number of hydrogen bonds between PUGNAc and VcNagZ, HsHexB, and hOGA exceeded that between N-valeryl-PUGNAc, EtBuPUG, and these three enzymes. These results provide the basis for the different potencies of PUGNAc, N-valeryl-PUGNAc, and EtBuPUG against the three enzymes.

Relative to the inhibitory activity of the PUGNAc derivatives, selectivity is a more attractive issue for exploration. PUGNAc binds well to VcNagZ, HsHexB, and hOGA; therefore, it displays a lack of selectivity. Upon increasing the size from N-acetyl to the N-valeryl group, N-valeryl-PUGNAc, possesses 667- and 121-fold selectivity for VcNagZ over HsHexB and hOGA, respectively. Further increasing the size of the substituent, EtBuPUG bearing a larger 2-isobutamido, shows 968- and 109-fold selectivity for VcNagZ over HsHexB and hOGA. The reason for these significant selectivities was shown by the MD simulations results. Firstly, the active site architectures of VcNagZ, HsHexB, and hOGA were found to be sufficiently different (Figure 3). The volumes of their active pockets were calculated using the software CASTp (http://sts.bioe.uic.edu/castp/index.html). The order of the sizes of their active pockets is VcNagZ (31.9 Å) > hOGA (25.8 Å) > HsHexB (18.2 Å). The VcNagZ has a large pocket with remarkable flexibility that can accommodate various inhibitors. HsHexB has a shallow substrate-binding pocket. hOGA has a medium pocket that more willingly than HsHexB accommodates rather voluminous substituents attached to the location of the substrate 2-acetamido group (Henrissat and Davies, 1997; Stubbs et al., 2007; Liu et al., 2012). Therefore, N-valeryl-PUGNAc may remain stable in the active pocket of VcNagZ and has different degrees of deviations in the active sites of HsHexB and hOGA. EtBuPUG is located well within the active pocket of VcNagZ and completely moved outside of HsHexB; however, it could still enter into the active pocket of hOGA in a special mode (Figure S4). These results reveal show the causes of selectivity from the structural characteristics of the enzymes.

Furthermore, interactions between inhibitors and binding residues of these nine systems showed apparent differences (Figure S5). N-valeryl-PUGNAc binds well into the pocket of VcNagZ by forming eight hydrogen bonds with Ala180, Arg73, and Asn285, but moves far from the pocket of HsHexB, which leads to interactions of four hydrogen bonds with Asp354, Asp452, and Glu491. The interactions between N-valeryl-PUGNAc and hOGA were also found to be weaker than those of N-valeryl-PUGNAc and VcNagZ. EtBuPUG remained firmly anchored in the open pocket of VcNagZ by forming five favorable hydrogen bond interactions with Asp65, Arg140, and His171. However, EtBuPUG cannot enter into the pocket of HsHexB, which results in activity loss with HsHexB, and only forms two hydrogen bond interactions with Arg211 and Glu491. These results may explain why EtBuPUG exhibits an 968-fold selectivity against VcNagZ over HsHexB.

Moreover, the differences in the catalytic mechanisms of VcNagZ, HsHexB, and hOGA also led to the selectivity of these PUGNAc derivatives. VcNagZ was first reported by Vocadlo et al. using a two-step double displacement mechanism (catalytic residues: Asp253 and Asp254) (Vocadlo and Withers, 2005). However, HsHexB (catalytic residues: Asp354 and Asp355) and hOGA (catalytic residues: Asp174 and Asp175) were reported to adopt a substrate-assisted mechanism, in which the 2-acetamido group of the non-reducing end sugar acts as a nucleophile, which results in the formation of a bicyclic oxazolinium intermediate (Terwisscha van Scheltinga et al., 1994; Tews et al., 1996; Mark et al., 2001). These differences may enable PUGNAc, N-valeryl-PUGNAc, and EtBuPUG to be selective to HsHexB/VcNagZ or hOGA/VcNagZ, especially the specificity of EtBuPUG to VcNagZ than HsHexB. This may also explain why the selectivity of these PUGNAc derivatives against hOGA/HsHexB is not large, and does not exceed 10-fold.

### Binding Free Energy Calculation

To further explore the cause of the inhibitory potency and selectivity of PUGNAc derivatives against VcNagZ, HsHexB, and hOGA, binding free energy analyses of the nine systems were performed by using MM/GBSA calculation methods. The results show that the absolute value of the binding free energy is consistent with the trend of inhibitory activities of compounds to the corresponding enzyme. As shown in Table 3, the value of the binding free energy (Δ*G*_TOT_) of PUGNAc is more negative than that of N-valeryl-PUGNAc and EtBuPUG, which means that PUGNAc has better inhibitory activities against the three tested enzymes than N-valeryl-PUGNAc and EtBuPUG. The binding free energy value of the HsHexB-EtBuPUG system is the maximum, indicating the worst inhibitory activity of EtBuPUG against HsHexB. Besides, experimental inhibition constants K_i_ (in μM) of PUGNAc derivatives measured against VcNagZ, HsHexB and hOGA (Macauley et al., 2005; Stubbs et al., 2006, 2007) were converted into log unit (pK_i_ = -LogK_i_). We found the experimental binding affinities (pK_i_) is in good agreement with the binding free energies calculated using MM/GBSA method results, and a linear correlation was obtained that yielded a good correlation coefficie (*R*^2^ = 0.895) (Figure S6).

**Table 3:** The binding free energy calculated by using MM/GBSA methods (kcal mol^−1^).

	**PUGNAc(1)**			**N-valeryl-PUGNAc(2)**			**EtBuPUG(3)**		
	**VcNagZ**	**HsHexB**	**hOGA**	**VcNagZ**	**HsHexB**	**hOGA**	**VcNagZ**	**HsHexB**	**hOGA**
**Δ*****E***_**VDW**_	−27.06	−30.88	−31.13	−30.66	−33.17	−36.21	−40.9	−38.84	−40.84
**Δ*****E***_**ELE**_	−83.81	−75.03	−69.94	−71.38	−50.54	−66.12	−60.44	−59.63	−65.04
**Δ*****G***_**SA**_	−6.76	−5.01	−5.95	−5.19	−4.33	−5.78	−5.71	−5.3	−6.3
**Δ*****G***_**GB**_	81.77	75.27	72.67	75.65	66.38	82.23	78.04	91.95	90.77
**Δ*****G***_**TOT**_	−35.86	−35.65	−34.35	−31.58	−21.66	−25.88	−29.01	−11.82	−21.41
**STD^a^**	3.19	3.52	3.36	3.68	2.81	2.90	2.78	2.89	2.53
**pK**_**i**_	1.32	1.44	1.34	0.48	−2.34	−1.6	−0.49	−3.48	−2.53

a *The standard deviation of the binding energies*.

Furthermore, four individual energy components (Δ*E*_vdw_, Δ*E*_ele_, Δ*G*_SA_, and Δ*G*_GB_) were carefully analyzed to better understand the energy terms with the greater impact on binding affinity. The van der Waals (Δ*E*_vdw_) and electrostatic (Δ*E*_ele_) contributions are highly favorable for the inhibitor binding to enzymes, and the electrostatic interactions exceed the van der Waals interactions. The data exhibited here are consistent with the previously reported electrostatic environment in which the active pocket of glycosidase is beneficial for the elimination of barriers (Passos et al., 2011). Moreover, the order of van der Waals interactions relative to the specific enzyme was EtBuPUG > N-valeryl-PUGNAc > PUGNAc, which matches the size sequence of the compound. The larger substituent at the 2-position of these compounds maybe closer to the hydrophobic region of the protein. The nonpolar desolvation energies (Δ*G*_SA_) among these nine systems are not much different and range between −4.33 and −6.76 kcal mol^−1^. However, the polar contributions of desolvation (Δ*G*_GB_) are very unfavorable for the binding of PUGNAc derivatives and these enzymes, which could offset their electrostatic interaction. Thus, the nonpolar interactions (Δ*E*_vdw_ + Δ*G*_SA_) in the system are more favorable for the binding of ligands to receptors.

### Decomposition Analysis of the Binding Free Energies

**Figure 4:**
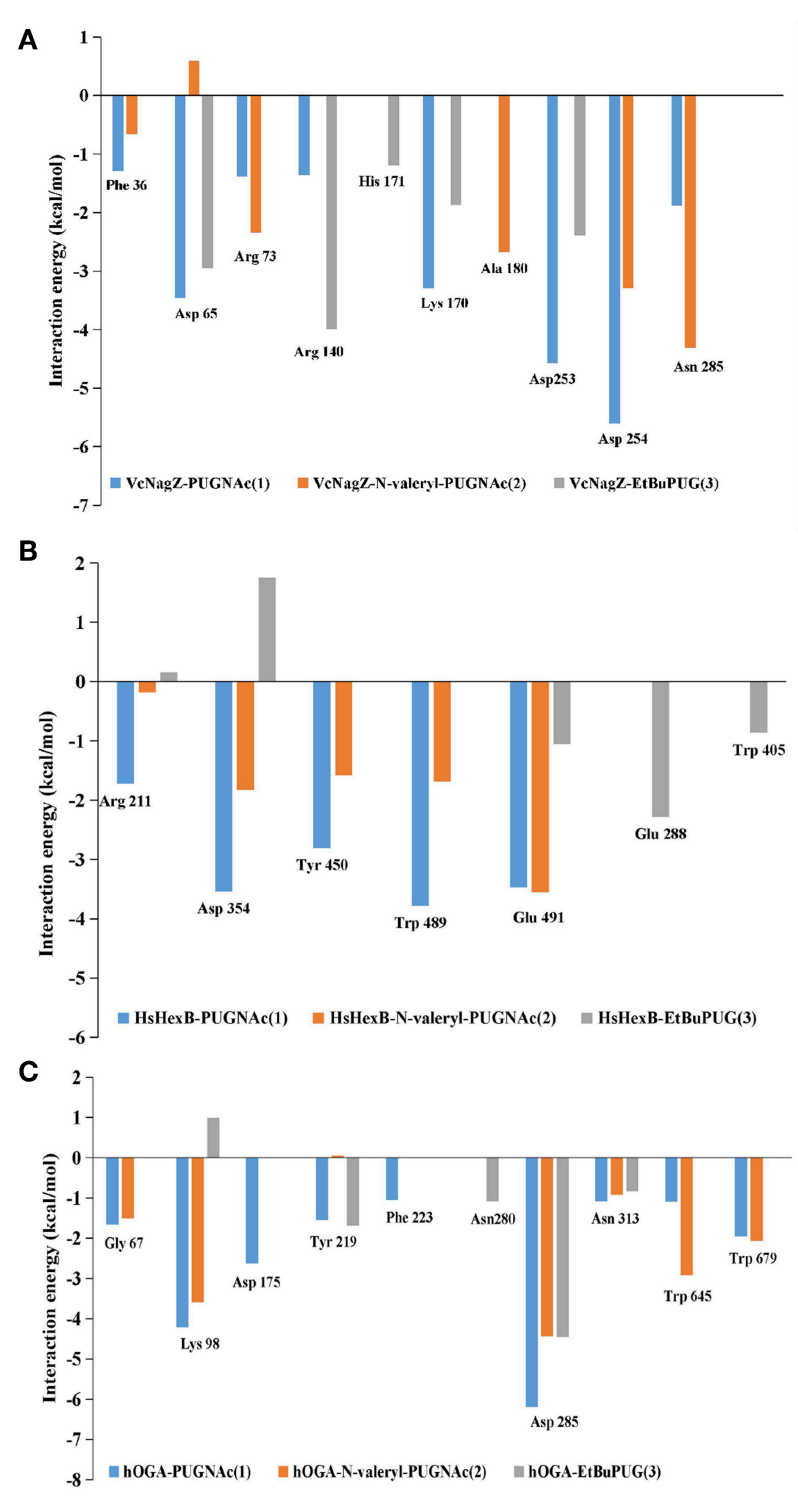
Comparison of key contributors of VcNagZ **(A)**, HsHexB **(B)**, and hOGA **(C)** with PUGNAc, N-valeryl-PUGNAc, and EtBuPUG interactions.

To obtain a detailed quantitative analysis of the contribution of each residue to VcNagZ, HsHexB, and hOGA with PUGNAc, N-valeryl-PUGNAc, and EtBuPUG interactions, MM/GBSA free energy decomposition analyses were performed to decompose the total binding free energies into residues. Residues near 4 Å of the ligands are used to calculate the decomposition free energy, and the key residue contributions of VcNagZ, HsHexB, and hOGA against the PUGNAc, N-valeryl-PUGNAc, and EtBuPUG, respectively, are shown in Figure 4.

In the VcNagZ-inhibitor systems, the differences in interactions are mainly reflected in the six residues, Arg140, Lys170, Ala180, Asp253, Asp254, and Asn285 (Figure 4A). Among these, the interactions of PUGNAc with the key residues Lys170, Asp253, and Asp254 are stronger than those of N-valeryl-PUGNAc and EtBuPUG. In particular, PUGNAc formed the strongest interaction with catalytic residue Asp253 and Asp254 (−4.57 and −5.61 kcal mol^−1^), while N-valeryl-PUGNAc only bound to Asp254 with moderate interaction (−3.29 kcal mol^−1^) and EtBuPUG only bound to Asp253 with lesser interaction (−2.39 kcal mol^−1^). These results could explain why the inhibitory activity of PUGNAc against VcNagZ was better than that of N-valeryl-PUGNAc and EtBuPUG. In addition, the movement of N-valeryl-PUGNAc in the VcNagZ pocket moved it closer to Ala180 and Asn285, thus forming more favorable interactions with them (−2.68 and −4.31 kcal mol^−1^). Similarly, the movement of EtBuPUG in the active pocket brings its pyranose closer to Arg140, resulting in greater interaction (−4.0 kcal mol^−1^).

In the HsHexB-inhibitor systems, distinct differences exist in the five residues, Arg211, Asp354, Tyr450, Trp489, and Glu491. Most of these residues have more favorable interactions with PUGNAc (−1.72, −3.54, −2.80, −3.78, and −3.47 kcal mol^−1^) than with N-valeryl-PUGNAc (−0.18, −1.83, −1.59, −1.69, and −3.55 kcal mol^−1^), respectively. It is worth noting that a stronger interaction exists between N-valeryl-PUGNAc and Glu491 than between PUGNAc and EtBuPUG, which means that N-valeryl-PUGNAc moved closer to Glu491 in the active pocket. EtBuPUG only showed weak interaction with the key residue Glu491(−1.06 kcal mol^−1^), but interacted with residues Glu288 and Trp405 (outside of the pocket). This is in accordance with the order of inhibitory activities of these PUGNAc derivatives against HsHexB.

In the hOGA-inhibitor systems, interactions between PUGNAc and residues Gly67, Lys98, Phe223, Asp285, and Asn313 of hOGA are more favorable than N-valeryl-PUGNAc and EtBuPUG. N-valeryl-PUGNAc has stronger interactions with Gly67, Lys98, Asn313, Trp645, and Trp679 than EtBuPUG. These results may explain why the order of activity against hOGA was PUGNAc > N-valeryl-PUGNAc > EtBuPUG. Furthermore, the apparent differences between these three inhibitors and hOGA were mainly identified on the catalytic residue Asp175. Only PUGNAc possesses a strong interaction with the value of−2.62 kcal mol^−1^. N-valeryl-PUGNAc and EtBuPUG could not interact with Asp175, resulting in lower inhibitory activity.

The decomposition analysis of the binding free energies explained why the inhibitory activities of N-valeryl-PUGNAc and EtBuPUG against VcNagZ was better than those of hOGA and HsHexB. The reason is that N-valeryl-PUGNAc and EtBuPUG cannot form an important binding interaction with the key residues Asp175 (hOGA) and Asp354 (HsHexB) in the active pockets. The contribution of the catalytic residues Asp253 and Asp254 (VcNagZ), Asp354 (HsHexB), and Asp175 (hOGA) in the enzyme-inhibitor systems were important for distinguishing the activity of these inhibitors.

## Conclusion

A computational approach that combines molecular docking, and MD simulations was employed to provide insights into the activity and selectivity of PUGNAc derivatives against GH3, GH20, and GH84 β-N-acetyl-D-hexosaminidases (VcNagZ, HsHexB, and hOGA). Firstly, the molecular docking combined with 20 ns MD simulations were performed to investigate the differences of these three enzyme active pocket architectures as well as the possible inhibitory patterns of the nine inhibitor-enzyme systems. The results show that the best-known inhibitor PUGNAc is located well at the active pockets of all three enzymes; therefore, it lacks selectivity. When the size of 2-acetyl of the sugar ring of PUGNAc is enlarged, the result is selectivity despite a degree of decrease in activity. Specifically, N-valeryl-PUGNAc, bearing a larger line 2-valeryl group, could be accommodated in the pockets of VcNagZ and hOGA. In particular, EtBuPUG, with the largest branched 2-isobutamido group, could only bind well to the VcNagZ pocket but was completely removed from the active pocket of HsHexB, thus lacking interactions with HsHexB. These distinctive differences in binding modes may lead to EtBuPUG exhibiting 968-fold selectivity to VcNagZ over HsHexB.

Analyses of MM/GBSA free energy calculations and MM/GBSA free energy decomposition demonstrate that the predicted energy of the PUGNAc-enzymes complexes is more favorable than that of the N-valeryl-PUGNAc-enzyme and the EtBuPUG-enzyme systems. Moreover, the van der Waals and electrostatic contributions are favorable to the combination of PUGNAc derivatives with the three enzymes. Furthermore, the catalytic residues Asp253 (VcNagZ), Asp254 (VcNagZ), Asp354 (HsHexB), and Asp175 (hOGA) are the most important residues for distinguishing the potential of the related inhibitors.

This is the first systematic study of the possible binding mechanisms for the dynamic interaction of GH3, GH20, and GH84 β-N-acetyl-D-hexosaminidases with inhibitors in solution. It is more in line with the interaction between drugs and target enzymes in the body. These computational study results indicate that positively charged functional groups (to bind to residue Asp) as well as an aromatic group at suitable positions of inhibitors would be helpful to improve the inhibitory potency against β-N-acetyl-D-hexosaminidases. On the other hand, the selectivity of inhibitors is mainly related to the pocket size of the enzymes. The bulky and branched group at the position of the 2-acetamido of the substrates would be beneficial for the selectivity against GH3 β-N-acetyl-D-hexosaminidases over GH20 and GH84 β-N-acetyl-D-hexosaminidases. Therefore, selectivity to different GH β-N-acetyl-D-hexosaminidases could be achieved by regulating the size of the group at this position. In summary, this work provides considerable structurally guided improvements for the development of novel, potent, and specific β-N-acetyl-D-hexosaminidases inhibitors.

## Author Contributions

The manuscript was written through contributions of all authors. All authors have given approval to the final version of the manuscript.

### Conflict of Interest Statement

The authors declare that the research was conducted in the absence of any commercial or financial relationships that could be construed as a potential conflict of interest.
