# Supplementary material for: Computational Studies on the Potency and Selectivity of PUGNAc Derivatives Against GH3, GH20, and GH84 β-N-acetyl-D-hexosaminidases
Source: Front Chem. 2019 Apr 12;7:235. doi: 10.3389/fchem.2019.00235 (PMC6499197; doi:10.3389/fchem.2019.00235)
Supplement: Supplementary file 1 [file Data_Sheet_1.pdf]

Computational Studies on the Potency and Selectivity of PUGNAc Derivatives against GH3, GH20, and GH84 β-N-acetyl-D-Hexosaminidases

Lili Dong, Shengqiang Shen, yefei Xu, Leng Wang, Ruirui Feng, Jianjun Zhang, Huizhe Lu**

Department of Applied Chemistry, College of Science, China Agricultural University, Beijing 100193, P.R. China

General method

The Sketch Molecule module and the Surflex-Dock algorithm in the The SYBYL 7.3 software package (Tripos Associates., 2006) were used to the modeling of small moleculars and the research of molecular docking. Density functional theory (DFT) in Gaussian 16 (Frisch et al., 2016) was employed to optimize the inhibitors. The MD simulations, binding free energy calculations and free energies decomposition of enzyme-inhibitor systems were studied using Amber14 program (Case et al., 2014).

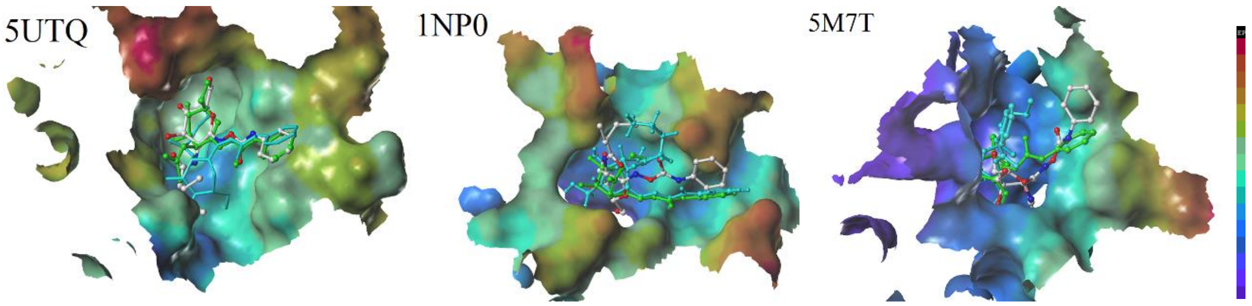

Figure S1. Electrostatic potential distribution in active pocket of VcNagZ (PDB ID:5UTQ), HsHexB (PDB ID:1NP0) and hOGA (PDB ID:5M7T). PUGNAc (green), N-valeryl-PUGNAc(blue) EtBuPUG(atom_type) were represented by a ball and stick.The charge from positive to negative was showed as different colors: from red to blue.

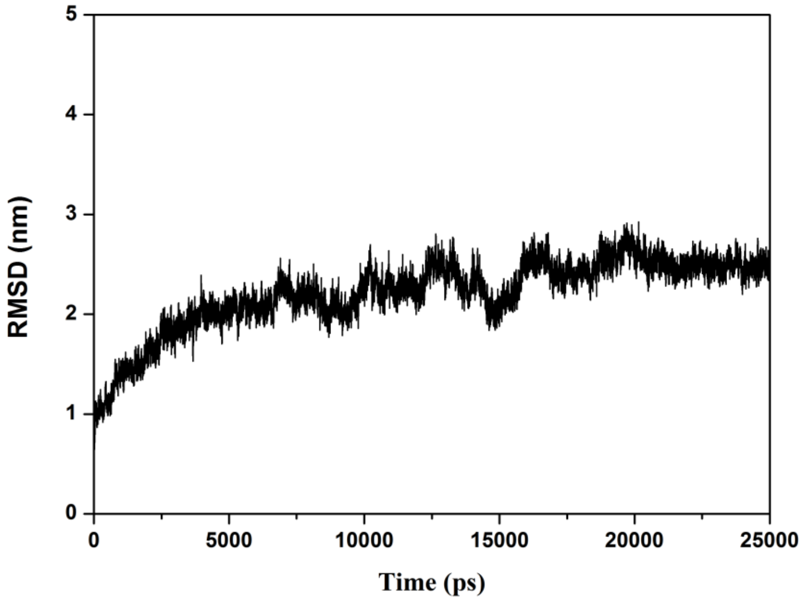

Figure S2. RMSD changes of the VcNagZ-**2** complex during MD simulations of 25 ns.

To analyze the local stability of the binding site, the RMSD values of inhibitors and the residues near 4 Å of the inhibitors were calculated and ultimately maintained around 1.1-2.6 Å for the nine simulated systems.

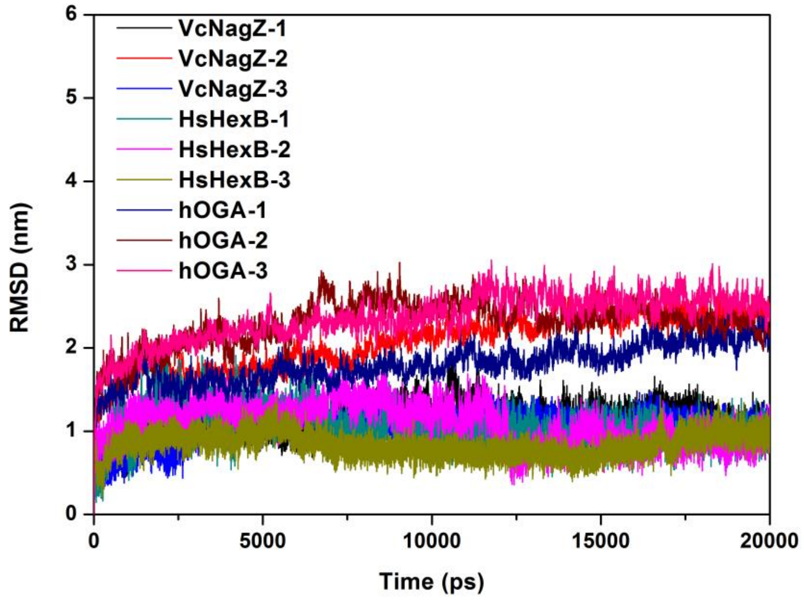

Figure S3. RMSD changes of the residues near the 4 Å of the inhibitors and inhibitors.

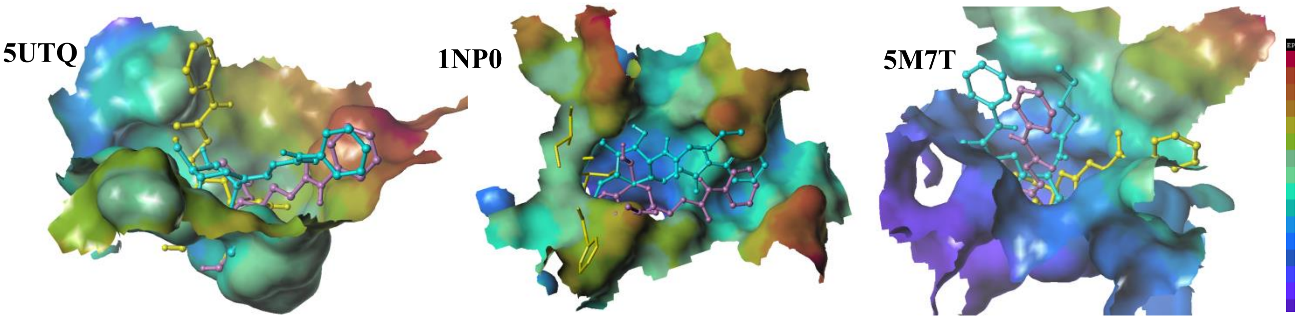

Figure S4. Electrostatic potential distribution in active pocket of VcNagZ (PDB ID:5UTQ), HsHexB (PDB ID:1NP0) and hOGA (PDB ID:5M7T). PUGNAc (pink), N-valeryl-PUGNAc(cyan) EtBuPUG(yellow) were represented by a ball and stick.The charge from positive to negative was showed as different colors: from red to blue.

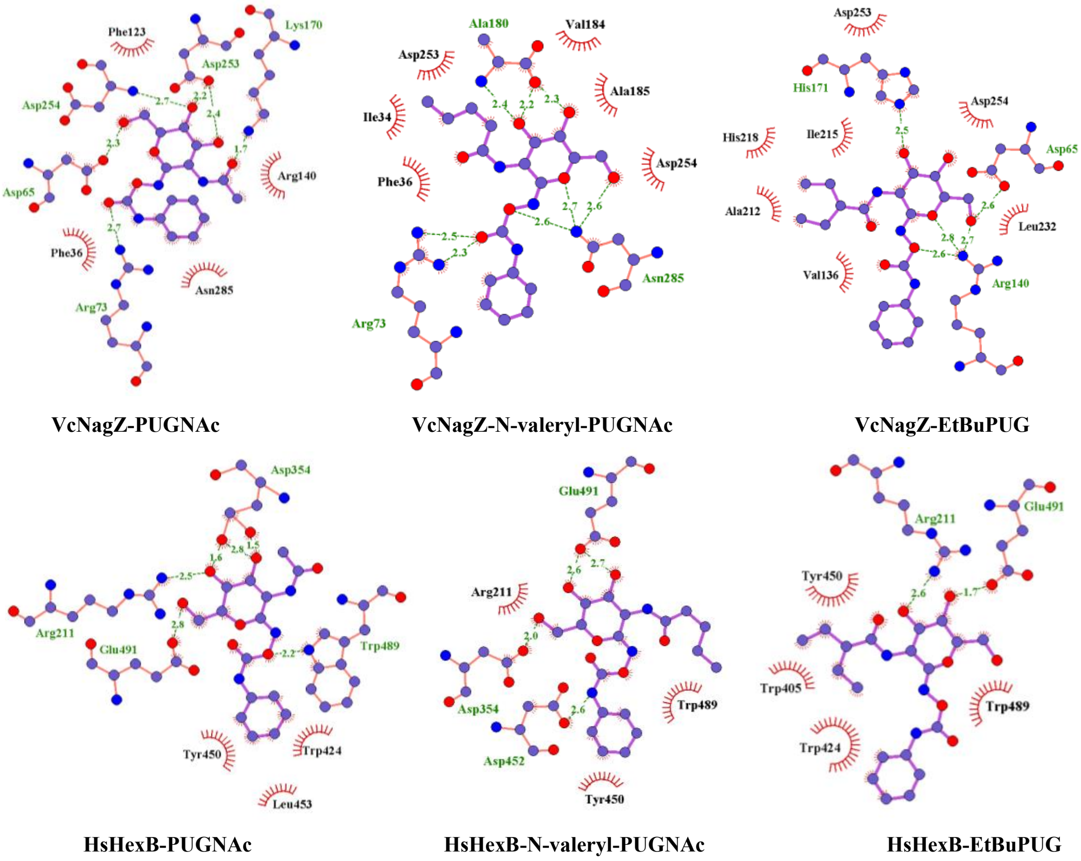

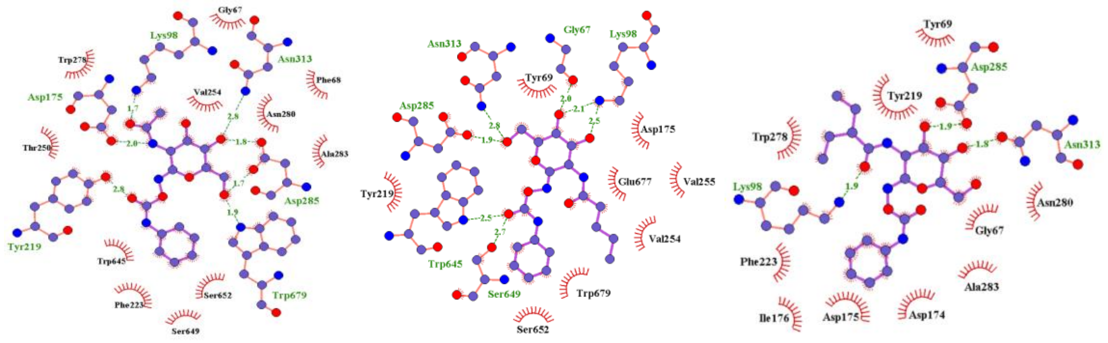

hOGA-PUGNAc **hOGA-N-valeryl-PUGNAc** **hOGA-EtBuPUG**

Figure S5. 2D Presentation of compounds PUGNAc, N-valeryl-PUGNAc and EtBuPUG in the active of VcNagZ, HsHexB and hOGA, respectively.

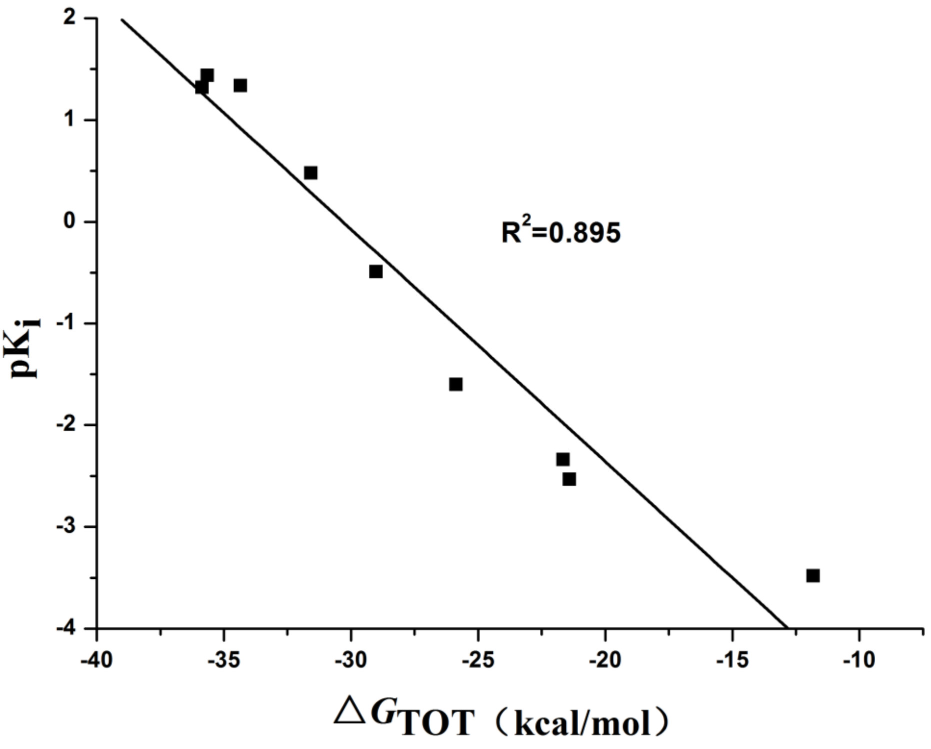

Figure S6. Relationship between the experimental pK_i and the calculated binding free energies of PUGNAc derivatives against VcNagZ, HsHexB and hOGA.

Reference

Case, D. A., Babin, V., Berryman, J. T., Betz, R. M., Cai, Q., Cerutti, D.S., Cheatham, T. E., Darden, T. A.,Duke, R.

E., Gohlke, H., Goetz, A.W., Gusarov, S., Homeyer, N., Janowski, P., Kaus, J., Kolossváry, I., Kovalenko, A., Lee, T.S., LeGrand, S., Luchko, T., Luo, R., Madej, B., Merz, K.M., Paesani, F., Roe, D.R., Roitberg, A., Sagui, C., Salomon-Ferrer, R., Seabra, G., Simmerling, C.L., Smith, W., Swails, J., Walker, R.C., Wang, J., Wolf, R.M., Wu, X., Kollman. P.A. (2014). AMBER 14, University of California, San Francisco.

Frisch, M. J., Trucks, G. W., Schlegel, H. B., Scuseria, G. E.,. Robb, M. A., Cheeseman, J. R., Scalmani, G., Barone, V., Petersson, G. A., Nakatsuji, H., Li, X., Caricato, M., Marenich, A. V., Bloino, J., Janesko, B. G., omperts, R., Mennucci, B., Hratchian, H. P., Ortiz, J. V., Izmaylov, A. F., Sonnenberg, J. L., D. Williams-Young, D., Ding, F., Lipparini, F., Egidi, F., Goings, J., Peng, B., Petrone, A., Henderson, T., Ranasinghe, D., Zakrzewski, V. G., Gao, J., Rega, N., Zheng, G., Liang, W., Hada, M., M. Ehara, M., Toyota, K., Fukuda, R., Hasegawa,J., Ishida, M., Nakajima, T., Honda, Y., Kitao, O., Nakai, H., Vreven, T., Throssell, K., Montgomery, J. A., Vreven, T. Jr., Peralta, J. E., Ogliaro, F., Bearpark, M. J., Heyd, J. J., Brothers, E. N., Kudin, K. N., Staroverov, V. N., Keith, T. A., Kobayashi, R., Normand, J., Raghavachari, K., Rendell, A. P., Burant, J. C., Iyengar, S. S., Tomasi, J., Cossi, M., Millam,J. M., Klene, M., Adamo, C., Cammi, R., Ochterski, J. W., Martin, R. L., Morokuma, K., Farkas, O., Foresman, J. B., Fox, D. J. (2016). Gaussian 16, Revision A.03; Gaussian, Inc.: Wallingford, CT.

Tripos Associates. (2006). Sybyl 7.3. Tripos Associates, St. Louis.
